# Degree of Actinic Elastosis Is a Surrogate of Exposure to Chronic Ultraviolet Radiation and Correlates More Strongly with Cutaneous Squamous Cell Carcinoma than Basal Cell Carcinoma

**DOI:** 10.3390/life13030811

**Published:** 2023-03-17

**Authors:** Konstantin Drexler, Hans Drexler, Sigrid Karrer, Michael Landthaler, Sebastian Haferkamp, Florian Zeman, Mark Berneburg, Dennis Niebel

**Affiliations:** 1 Department of Dermatology, University Hospital Regensburg, 93053 Regensburg, Germany; 2 Institute for Vocational, Social and Environmental Medicine, University Hospital Erlangen, 91054 Erlangen, Germany; 3 Center for Clinical Studies, University Hospital Regensburg, 93053 Regensburg, Germany

**Keywords:** actinic elastosis, non-melanoma skin cancer, keratinocyte cancer, occupational disease, histopathology

## Abstract

(1) Background: Keratinocyte cancer (KC) is associated with exposure to ultraviolet (UV) radiation. However, data are controversial as to whether chronic UV exposure or high intermittent UV exposure are key drivers of carcinogenesis in cutaneous squamous cell carcinoma (cSCC) and basal cell carcinoma (BCC). Prolonged sun exposure of the skin causes photo-aging, which is associated with actinic elastosis, a condition characterized by the degeneration of elastin in the upper dermis, which is assessable via conventional histology. In this study, we aimed to compare the degree of actinic elastosis in different types of KC with regard to various patient characteristics. (2) Methods: We defined a semiquantitative score for the degree of actinic elastosis ranging from 0 = none to 3 = total loss of elastic fibers (basophilic degeneration). The extent was measured histometrically by two independent dermatohistopathologists in the immediate vicinity of 353 KC. The scores were merged and matched with tumor types (cSCC and BCC with subtypes), and clinical variables such as body site, sex and age. (3) Results: As expected, the degree of actinic elastosis correlated with age. However, it was significantly higher in cSCC compared to BCC irrespective of age, sex, body site and tumor subtypes. (4): Conclusions: Lifetime sun exposure may be estimated via routine histology using this scoring technique for actinic elastosis as a surrogate marker. cSCCs are more strongly associated with chronic UV exposure than BCCs, even in sun-exposed localizations such as the face.

## 1. Introduction

Ultraviolet (UV) light imposes major physical and biological effects on the skin. Depending on the wavelength, one can distinguish UV-A (320–400 nm), UV-B (280–320 nm) and UV-C (40–280 nm) [1]. As UV-C is almost completely absorbed in the atmosphere, typically, only the two former mentioned types of UV light reach the skin surface to exert biological effects. While UV-B is overwhelmingly absorbed in the epidermis, UV-A may penetrate the skin up to the dermis with respective effects on cells and connective tissue.

From a pathophysiological perspective, skin aging and carcinogenesis represent the most important consequences of UV light exposure of the skin. The loss and fragmentation of fibers in the dermis is an important aspect of external aging. It results in reduced skin elasticity and deep wrinkles. Other clinical signs of chronic UV exposure include, among others, cutis rhomboidalis nuchae, erythrosis interfollicularis nuchae and Morbus Favre–Racouchot [2]. Histologically, standard hematoxylin and eosin staining may visualize altered/elastotic extracellular material as basophilic degeneration; this is also called actinic elastosis [3]. The origin of the elastic material is not completely clear. It is assumed, that either the actinic stimulation of fibroblasts or the degradation of collagen fibers, or even a combination of both effects, are responsible for developing actinic elastosis [3]. Moreover, UV exposure renders the activity of melanocytes, resulting in uneven pigmentation and numerous lentigines [4]. However, these effects are not limited to chronic UV exposure but also occur in the course of high intermittent UV exposure [5].

A correlation between UV light exposure and the risk for certain types of skin cancer, including keratinocyte cancer (KC) (i.e., cutaneous squamous cell carcinoma (cSCC), basal cell carcinoma (BCC)) and melanoma was established decades ago [6]; the main drivers are cytosine–thymine transitions (desoxyribonucleic acid (DNA)-damage; C-T transition) [7] and the immunosuppressive effects of UV light dampening immune surveillance [8]. Intriguingly and counterintuitively, certain clinical manifestations related to chronic UV exposure were described to show a protective effect with regard to KC risk [9]. Clearly, ethnic origin, Fitzpatrick phototype, childhood UV exposure and other dermatologic conditions such as a history of acne must be taken into account regarding the lifetime risk to develop KC [10]. Notably, inherited genodermatoses may carry a dramatically increased risk of the development of KC including but not limited to xeroderma pigmentosum, oculocutaneous albinism, epidermodysplasia verruciformis (Lewandowsky–Lutz dysplasia), Rombo syndrome, Bazex–Dupré–Christol syndrome and nevoid basal-cell carcinoma syndrome (Gorlin–Goltz syndrome).

Up to now, it has remained unclear how exactly and why the risk of different entities of KC differs with regard to repetitive/ongoing high UV exposure (i.e., occupational/outdoor workers) compared to high intermittent exposure (i.e., recreational/indoor workers) and a combination of both. The risk for cSCC and its in situ precursor actinic keratosis (AK) strongly correlates with lifetime UV exposure reflected by soaring odds ratios starting with lifetime sun exposure of more than 100,000 h [11]. In Germany, cSCC and recurrent AK qualify as occupational diseases, assuming a substantial occupational contribution of the lifetime UV burden of at least 40% [12]. A population-based case–control study consistently described a two-fold increase for the risk of cSCC in highly exposed outdoor workers [13]. Studies investigating the connection between cumulative UV exposure and the risk for basal cell carcinoma (BCC) found deviating results, though. Even though BCCs tend to favor light-exposed body sites such as the face, occupational sun exposure was found to be protective in a large study, while high-intermittent UV exposure, e.g., during beach activities, led to a risk increase [11]. The odds ratio for the development of BCC reaches a plateau with 1000 h of sun exposure irrespective of up to a hundred-fold higher exposure (100,000 h). More recently, a Swedish population-based case–control study even described a shift of BCC incidence towards indoor workers such as dentists, legal workers and physicians when compared with farmers, foresters and gardeners [14]. Potential reasons that were discussed in the paper included the high socioeconomic status of the indoor workers that allowed them to spend more time with leisure activities accounting for high intermittent UV exposure. Outdoor workers in Nordic countries might cover more body parts in light of lower temperatures when compared to their counterparts in regions with a warmer climate, which could contribute to the effect seen in the study. Another case–control study included more than 200 individuals with BCC to assess clinical variables and the risk of sporadic BCC. Actinic elastosis was found more frequently in melanoma compared to BCC; cSCC were not included [15]. Whiteman et al. assumed divergent pathways in the development of malignant melanoma. One might be associated with sun exposure and the other with melanocytic proliferation [16]. In children, intermittent intense sun exposure was shown to play an important role in nevus development [17].

A meta-analysis published in 2013 described associations of BCC with actinic elastosis (unquantified), solar lentigines and telangiectasia with odds ratios of around 1.5, while the presence of more than ten AK showed the largest association with BCC [18]. The presence of numerous AKs is associated with chronic UV exposure; therefore, this might indirectly point towards an association of BCC development with chronic UV exposure. In line with this, a population-based case–control study described a two-fold increased risk of BCC in occupationally exposed areas compared to non-exposed individuals irrespective of tumor localization and Fitzpatrick phototype [19]. Interestingly, multivariate analysis did not reveal changes in BCC subtypes even though superficial BCCs commonly arise in non-sun-exposed localizations such as the trunk, whereas nodular BCCs most often occur on the face, neck and head. Moreover, the same research group published results of a large study with more than 800 BCC patients matched with healthy controls that established a tendency towards an occupational impact on the overall risk to develop BCC [20].

As indicated before, actinic elastosis might represent a variable directly associated with chronic UV exposure rather than intermittent UV exposure [21]. Indeed, it was proposed as a surrogate marker of chronic UV damage in 2020, which is accessible for exact quantification via histopathology [22]. Riegler et al. used a quantitative approach measuring the depth of actinic elastosis in corpses of young and elderly individuals compared to fetuses and found statistically significant differences; however, they did not include a semiquantitative dimension for the degree of actinic elastosis visible. In another study, image analysis was used to determine the amount of elastotic material adjacent to skin tumors. There was a 3–4 fold increase when compared to healthy skin; however, the authors did not include an analysis between different entities of KC [23]. In our perception, based on the aforementioned published data, it is paramount to distinguish between cSCC and BCC rather than subsuming both.

Therefore, the purpose of this study was to quantify actinic elastosis as a surrogate of cumulative UV-induced damage in the adjacent tissue of different subtypes of KC and to correlate the findings with age, sex, anatomic site and other clinical parameters. Given the rising incidence of both KC and melanoma, it is crucial to estimate the role of UV light in different tumor entities and to stratify which other clinical variables contribute most to carcinogenesis. Even though the discussion about the impact of chronic UV exposure may be extended to subtypes of cutaneous melanoma (e.g., lentigo maligna melanoma, nodular melanoma, superficial spreading melanoma, acral lentiginous melanoma) [24] and rare tumors such as Merkel cell carcinoma, atypical fibroxanthoma and pleomorphic dermal sarcoma [25], this study will focus on KC.

## 2. Materials and Methods

### 2.1. Patient Characteristics and Inclusion Criteria

First, we determined the number of histopathological slides in our academic research center in south-eastern Bavaria, Germany, with regard to the number of cases of KC (single-center study). In light of a rising overall incidence of KC in Germany, we compared the years 1999, 2009 and 2019 regarding the overall number of specimen and the number of cSCC and BCC, putting further emphasis on histological subtypes of BCC (superficial, nodular, sclerodermiform).

For the analysis of actinic elastosis, we randomly selected the most recent n = 400 cases of KC starting on 1 January 2021 with a proportion of n = 100 cSCC and n = 300 BCC (100 per histological subtype, i.e., superficial, nodular and sclerodermiform). Thirteen of cSCC, 2 of superficial BCC, 1 of nodular BCC and 56 of sclerodermiform BCC had to be excluded due to a failure in the quality of the histopathology. Clinical data including age, sex and body site were extracted from i.s.h.med software (Cerner Corporation, North Kansas City, MO, USA, run via SAP 6.0 software, SAP SE, Walldorf, Germany), which is used as hospital management software in our institution. Face, head, hands and dorsal forearms were defined as “UV-exposed” body sites.

### 2.2. Histopathological Assessment

Sections were processed according to standard protocol and stained with hematoxylin–eosin. The histological examination was performed independently by two experienced dermatopathologists (K.D. and D.N.). Specimen slides were sorted by date of excision and not by tumor types to allow objective measurement. To assess the width of actinic elastosis, the widest identifiable elastotic fibre/area with basophilic degeneration in proximity of tumor and absence of tumoral stroma was measured orthogonally from the stratum granulosum using a scale ocular. To assess the degree of actinic elastosis, a semiquantitative score was established as follows: (0 = absent, 1 = low: less elastotic material than regular fibers (collagenous and elastic), 2 = moderate: more elastotic fibers than regular fibers, 3 = strong: complete or near complete loss of normal fibers/homogenous basophilic zone). If their counts were consistent within a range of 1 point in the score and a range of 20% in measurement of the width of elastosis, the mean was calculated and used for subsequent analyses. The agreement between both raters was medium for the degree of AE (kappa = 0.334; 95%-CI: 0.27, 0.40) as well as for TEG (ICC = 0.463; 95%-CI: 0, 0.72), indicating that the mean of two raters is a good choice to obtain reliable values. Inconsistent results could be resolved using a discussion microscope. The width was multiplied with the semiquantitative score resulting in the newly defined tumor-associated elastosis grading score (TEG). Curettage material, punch biopsies and specimens without normal tissue in the vicinity were excluded from further analysis to allow sufficiently reliable results. In the end, a total of n = 328 specimen from n = 321 patients were included in the statistical analysis.

### 2.3. Statistical Analysis

Statistical tests were performed using IBM SPSS version 25 (Armonk, NY, USA) and MS Excel Professional Plus 2016 Version 16.0.5369.1000 (Microsoft Corp., Redmond, WA, USA). Age at diagnosis of BCC/cSCC was compared for the years 1999, 2009 and 2019 using Student’s *t*-test. Degree of actinic elastosis and TEG between BCC and cSCC was compared using Student’s *t*-test. To evaluate other clinical variables (sex, age at diagnosis, UV-exposed areas) we performed multiple regression and analysis of variance (ANOVA). Results were considered statistically significant for *p* ≤ 0.05.

### 2.4. Microscope and Digital Photography

Both dermatopathologists used an Olympus BX43 microscope (Olympus, Shinjuku, Japan) for their analyses. All photomicrographs were captured after slide scanning using a PreciPoint M8 microscope and scanner with ViewPointLight software version 1.0.0.9628 for imaging (PreciPoint GmbH, Freising, Germany); we refrained from digital enhancement. Figures were created using MS PowerPoint Professional Plus 2016 Version 16.0.4266.1001 (Microsoft Corp., Redmond, WA, USA).

## 3. Results

### 3.1. Patient Characteristics

First, we aimed to assess the trend in our institution (University Hospital Regensburg, Bavaria, Germany) with regard to the overall amount of specimens and number of KCs over the course of 20 years. The catchment area of our institution includes the urban area of Regensburg with a population of ~150,000 inhabitants and surrounding rural counties (>1,000,000 inhabitants) with a lower density of dermatologist specialists compared to the city [26].

#### 3.1.1. Clinical Characteristics of KC Patients in Regensburg/South-Eastern Bavaria over the Course of 20 Years

The overall amount of specimens sent to our institute for histopathological evaluation roughly doubled over the course of 20 years from 1999 to 2019 (increase from 18,121 to 34,588) (Table 1). However, the diagnoses of KCs increased disproportionally within the same time. In fact, the total number of BCCs rose more than 2.5-fold and the number of cSCCs rose almost 4-fold. Conclusively, the relative frequency of the given diagnoses increased, which means that in 2019 one in six specimens were KC, opposed to one in ten specimens in 1999.

The absolute number of KC, i.e., diagnosis of BCC and cSCC, rose disproportionally compared to the increase in specimens. The age at diagnosis of BCC or SCC remained similar: patients tended to be 4–7 years older in 2019 when compared to 1999.

Many dermatologists have the feeling that there is a trend towards a diagnosis of BCC in younger years, while cSCC remains a diagnosis of the elderly patient. Therefore, we analyzed the age of diagnosis of both tumor types over the course of time. There was no statistically significant change in the age of diagnosis for BCC and cSCC between 1999 and 2019 (*t*-test was used, *p* = 0.83, *p* = 0.78, respectively). The mean age of diagnosis of BCC rose from 66.1 to 70.9, which is only a few years earlier than cSCC with a mean age of diagnosis rising from 72.3 to 77.9. Moreover, the mean age of diagnosis of the youngest 100 patients with the diagnosis of BCC in a given year remained unchanged at around 40 years. As a comparison: the mean age of the general population in Germany was 40.8 in 1999 and 44.5 years in 2019 [27].

#### 3.1.2. Clinical Characteristics of Patients Included in the Histopathological Analysis

**Figure 1:**
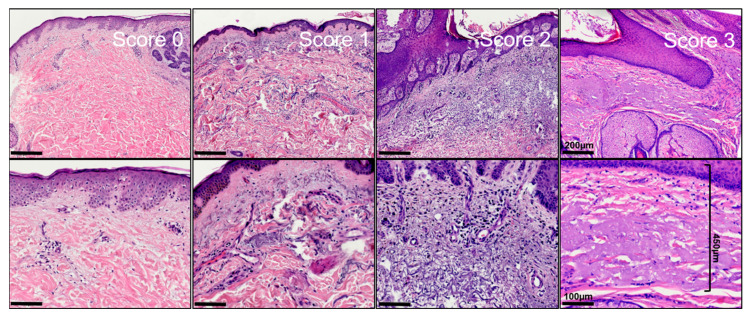
Exemplified histopathological assessment measurement of actinic elastosis and scoring (tumor-associated elastosis grading—TEG). The thickness/width of elastotic material was measured in the vicinity of the tumor in absence of tumoral stroma. Elastosis was scored as follows: 0 = absent, 1 = low: Less elastotic material than regular fibers (collagenous and elastic), 2 = moderate: More elastotic fibers than regular fibers, 3 = strong: Complete or near complete loss of normal fibers/homogenous basophilic zone.

To assess the degree of actinic elastosis in a meaningful and unbiased cohort of patients, we selected the 400 most recent specimens with a diagnosis of KC starting on 1 January 2021 with a ratio of 100 cSCCs and 300 BCCs. We chose this recent date to allow the best quality of slides and to obtain results comparable to the year 2019 (Figure 1). Moreover, BCCs were balanced for equal numbers of superficial, nodular and sclerodermiform BCCs. The clinical variables of the study cohort are depicted in Table 2.

In total, 328 slides from 321 patients were eligible for analysis and included in the study. The age of diagnosis differed between BCC and SCC by around 7 years. The proportion of tumors arising in UV-exposed body sites was higher in SCC compared to BCC even though both occurred in the majority of cases in association with UV-exposed areas. The mean thickness of actinic elastosis as well as TEG differed significantly between BCC and SCC, with SCC yielding higher numbers.

The majority of patients were male (59.9% for BCC and 68.0% for cSCC), and the mean age of the patients in this subsequent analysis closely resembled the figures found regarding the overall number of patients in 2019 (BCC: 71.3 compared to 70.9; cSCC: 78.2 compared to 77.9). The majority of KC was located in UV-exposed body sites as defined earlier; however, this applied to 55.8% of BCC but 76.3% of cSCC.

### 3.2. Histopathological Results—Thickness/Width of Actinic Elastosis in Regard to Age, Sex, Body Site and Tumor Type

**Figure 2:**
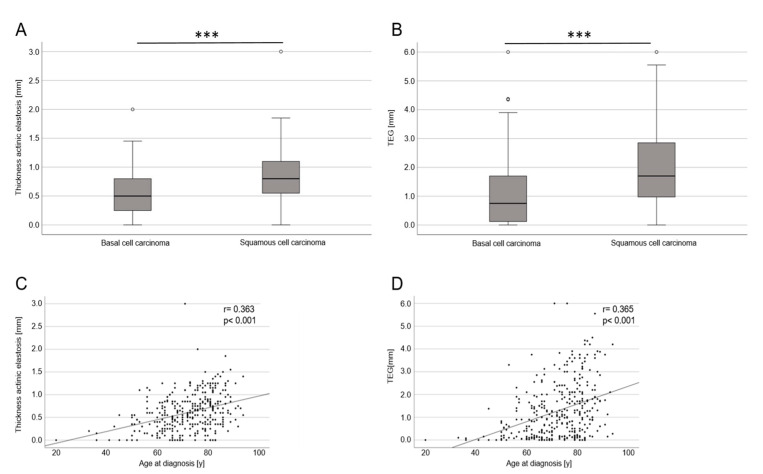
Mean thickness of actinic elastosis and TEG in the histological analysis comparing BCC with SCC and age of diagnosis of the patients included. (**A**) Boxplot diagram of the mean width of actinic elastosis demonstrating differences between BCCs overall and cSCC (*p* < 0.001; *** stands for *p* < 0.001). (**B**) Boxplot diagram of TEG (width × semiquantitative score) demonstrating differences between BCCs overall and cSCC (*p* < 0.001; *** stands for *p* < 0.001). (**C**) Point scatter plot demonstrates a significant correlation (*p* < 0.001) between mean width of actinic elastosis and age of tumor diagnosis. (**D**) Point scatter plot demonstrates a significant correlation (*p* < 0.001) between TEG and age of tumor diagnosis.

Both the mean width of actinic elastosis and the TEG increased with age (*p* < 0.001) (Figure 2C,D). This applied equally for both females and males. Both the mean thickness of actinic elastosis and the TEG were significantly higher in cSCC than in BCC (*p* < 0.001). As stated earlier, superficial BCCs typically arise on the trunk, which was considered non-UV-exposed. Omitting superficial BCCs from the analysis did not change the results though; a statistically significant difference remained between the tumor entities.

**Figure 3:**
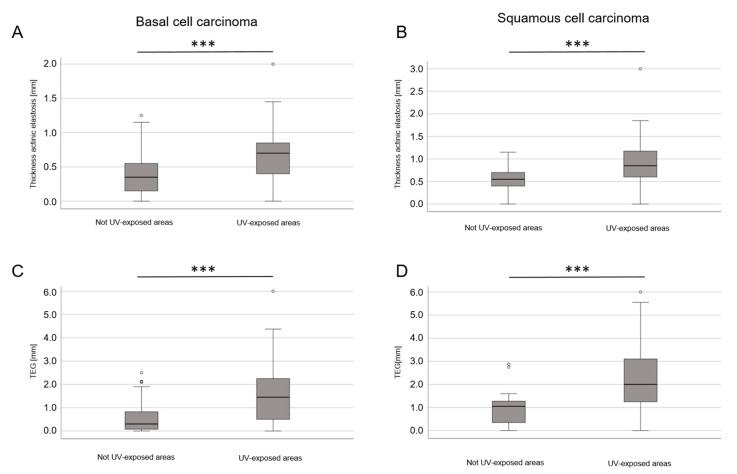
Mean thickness of actinic elastosis and TEG in the histological analysis comparing non-UV-exposed areas with UV-exposed areas for BCCs overall and cSCC. (**A**,**B**) Boxplot diagram of the mean width of actinic elastosis demonstrating differences between non-UV-exposed areas and UV-exposed areas in BCCs overall and cSCC (*p* < 0.001; *** stands for *p* < 0.001). (**C**,**D**) Boxplot diagram of TEG (width × semiquantitative score) demonstrating differences between non-UV-exposed areas and UV-exposed areas in BCCs overall and cSCC (*p* < 0.001; *** stands for *p* < 0.001).

There was also a correlation between the width of actinic elastosis and TEG in UV-exposed areas (face, head, hands and dorsal forearms) compared to non-exposed areas (Figure 3). The differences in the width of actinic elastosis (*p* = 0.006) and TEG (*p* < 0.001) between BCC and cSCC remained statistically significant when age and UV-exposed sites were included in the calculation.

A significant effect on the width of actinic elastosis was found for UV-exposed areas (regression coefficient 0.266), the diagnosis of cSCC (regression coefficient 0.128) and age at diagnosis (0.009). Moreover, for TEG, a correlation was seen for all parameters (*p* < 0.001). UV-exposed localization showed an effect (regression coefficient 0.968) as well as diagnosis of cSCC (regression coefficient 0.446) and age at diagnosis (regression coefficient 0.024; Table 3).

**Figure 4:**
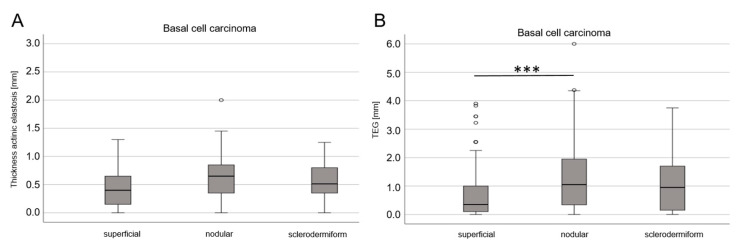
Comparison of width of actinic elastosis and TEG in subtypes of BCC. (**A**) Boxplot diagram of width of actinic elastosis in superficial, nodular and sclerodermiform BCC; the differences did not reach statistical significance. (**B**) Boxplot diagram of TEG in superficial, nodular and sclerodermiform BCC; the difference between superficial BCC and nodular BCC was significant (*p* < 0.001; *** stands for *p* < 0.001). There were no significant differences in multiple regression including UV-exposed localizations and age at diagnosis.

Even though superficial BCCs tend to arise in non-UV-exposed localizations such as the trunk, the width of actinic elastosis did not differ statistically significantly between the three analyzed subtypes (Figure 4). The same result was found for TEG when comparing all three tumor subtypes. There was only a significant difference in TEG between superficial BCC and nodular BCC (*p* = 0.041).

## 4. Discussion

The most important finding of our study is that the mean width and the degree of actinic elastosis, which was measured using the newly established TEG score, differed between BCCs and cSCCs irrespective of age during diagnosis and localization of the tumor (UV-exposed vs. non-UV-exposed body sites). This is of relevance as these skin tumors are commonly referred to as KC or “Non-melanoma skin cancer” (NMSC), which is too imprecise considering that the biology and the malignant potential of these tumors are different. Many studies do not sufficiently distinguish between BCC and cSCC [28] and they are even subsumed with a singular ICD-10 code (C44.x). As the TEG score correlates directly with the age at diagnosis, we assume that it works as an indicator for chronic exposure to UV radiation. Our results point towards a greater correlation between chronic UV exposure and the incidence of cSCC when compared to the incidence of BCC, which is in line with the previous literature as outlined in the Introduction.

The number of specimens in our institution increased more between 1999 and 2009 than between 2009 and 2019. There might be various reasons for this. First, during the phase of expansion, the number of senders was variable and so was the number of specimens sent by the individual senders. A larger proportion of tumors in the entity of the specimens, especially cSCC, might point towards a prioritization towards the excision of malignant tumors rather than benign lesions (e.g., cysts) in the respective dermatological practices. On the other hand, these numbers might merely reflect the rising incidence of KC in an aging German society. Almaani et al. described an increasing incidence of KCs in Jordan over 16 years, although this was not significant [29]. A rising incidence for KCs has been described in Germany as well, but this has not differed between cSCC and BCC [30]. Our findings are limited, as we only looked at the histopathological findings of a single center. Moreover, we did not correlate the catchment area or population data of the patients. It might be interesting to correlate our findings with other dermatohistopathological centers, as we assume that our colleagues will see a similar trend.

Another interesting finding of our study is that the age of diagnosis of BCC did not change in south-eastern Bavaria over the course of the last 20 years. This finding deviates from another single-center study performed in a middle-south Italian population who found a rising incidence of BCC in patients younger than 35 [31]. In a previous Australian study analyzing 3885 cases of BCC, patients with superficial BCC tended to be younger than patients with nodular and sclerodermiform BCC [32]. Similar findings were generated by an Italian group who found that younger patients tend to develop superficial BCC on the trunk, while elderly patients are more prone to developing nodular or sclerodermiform BCC on the face and head (UV-exposed areas) [33]. These findings are in accordance with the results of Pelucchi et al. who showed a correlation between occupational exposure to sunlight and the diagnosis of nodular BCC. Interestingly, they did not see this effect for superficial BCC [34]. Hence, we could not find new clues for a stronger association of nodular and sclerodermiform BCCs to UV exposure when compared to superficial BCCs. Yet, our results did show a respective trend, reflected in a higher degree of actinic elastosis in nodular BCC compared to superficial BCC. Including more cases into the analysis might result in statistically significant differences between the three tumor types. Notably, from a histopathological perspective, there are numerous other rare subtypes of BCC (e.g., metatypical BCC), which were not addressed in our analysis. As some BCCs even show squamatization, these peculiar tumors might resemble a third group in between classical BCC and cSCC, which will be interesting to analyze in the future. At this point, the exact role of chronic UV exposure remains controversial regarding the risk of developing different subtypes of BCC.

To integrate our findings, it is worthwhile considering studies dealing with melanoma and actinic elastosis. There is one Australian study that used a semiquantitative approach similar to ours to distinguish melanomas related to chronic sun exposure opposing nevus-associated melanomas [35]. The authors established a strong correlation of actinic elastosis with age, similar to our results. The same association was found in another study examining actinic elastosis in more than 1000 melanomas [36]. It is noteworthy that actinic elastosis may bias the interpretation of melanocytic lesions as it is a diagnostic criterion in dysplastic lesions [37]. The occurrence of acral lentiginous melanoma does not correlate with UV exposure as shown earlier [38]; the impact of UV exposure on other melanoma subtypes is less well-defined though. TEG might be used as a new tool to systematically analyze differences in the degree of actinic elastosis in subtypes of malignant melanoma. Lentigo maligna and its invasive counterpart lentigo maligna melanoma should be compared to other subtypes, as histopathological features include signs of chronic UV damage such as actinic elastosis and epidermal atrophy [39]. This will be addressed in ongoing studies of our research group.

Our study has several limitations. Firstly, there might be a regional bias due to the monocentric character of the analysis. Bavaria is in the southern part of Germany and the climate conditions differ from other German regions. Secondly, we do not have further socioeconomic information of the patients and no information regarding sun exposure. Therefore, we cannot draw conclusions regarding occupational and recreational attributions of UV exposure. Thirdly, the mere presence of elastotic material is only an indirect surrogate of chronic UV exposure, but the significant differences between UV-exposed body sites and non-UV-exposed body sites indicate a direct correlation. Darker skin types with larger amounts of melanin in the skin might tend to develop less elastotic material with the same cumulative dose of UV radiation. Other contributing factors to cutaneous carcinogenesis such as immunosenescence may not be assessed via conventional histology. Another factor that could not be assessed in this study was the attribution of human papilloma virus infections, which is a risk factor for cSCC but not BCC [40]. We propose to integrate the patients’ detailed medical history and the aforementioned variables with our newly established TEG in prospective studies to assess other clinical factors associated with KC development.

Due to a rising incidence of KC in an aging society, occupational risks related to chronic UV exposure must be taken into account to allow evidence-based prevention [41]. In Germany, cSCC is legally recognized as an occupational disease if certain criteria are fulfilled [42,43]. Currently, the establishment of BCC as an occupational disease is under debate [44]. Therefore, even more studies are needed to integrate the contribution of various factors and modes of sun exposure to KC development, especially BCC and subtypes. Education of the public about photoprotection and measurements against occupational skin cancer appear mandatory [45]. Artificial sources of UV light such as sunbeds should also be reappraised critically and must be part of the equation.

In conclusion, our study shows no differences in the age at diagnosis for BCC and cSCC over the course of the last two decades in our research center in south-eastern Bavaria. Interestingly, we could describe a stronger correlation of cSCC with chronic UV exposure compared to BCC assessed via conventional histology using a newly defined grading system.

## Figures and Tables

**Table 1:** Clinical characteristics of KC patients over the course of 20 years from 1999 to 2019.

	1999	2009	2019
Total number of specimen	18,121	32,191	34,588
Total number of BCC	1656	3240	4477
Diagnosis of BCC (%)	9.14	10.06	12.94
Age at diagnosis BCC (y)	66.1 (±13.3)	67.8 (±13.0)	70.9 (±12.4)
Age at diagnosis BCC (100 youngest) (y)	40.2 (±6.4)	37.1 (±6.6)	40.2 (±6.0)
Total number of SCC	220	352	853
Diagnosis of SCC (%)	1.21	1.09	2.47
Age at diagnosis SCC (y)	72.3 (±14.0)	75.7 (±10.8)	77.9 (±9.5)

**Table 2:** Clinical characteristics of patients included in the histopathological analysis.

	BCC	SCC	*p*
Sex	male = 145 (60.2%) female = 96 (39.8%)	male = 60 (69.0%) female = 27 (31.0%)	
Age at diagnosis (y)	69.7 (±11.6)	78.1 (±9.0)	≤0.001
UV-exposed body site	121 (50.2%)	65 (74.7%)	
Mean thickness of actinic elastosis (µm)	0.53 (±0.37)	0.81 (±0.45)	≤0.001
TEG (µm)	1.05 (±1.09)	1.91 (±1.33)	≤0.001

**Table 3:** Multiple regression showing the correlation between thickness of actinic elastosis and TEG. Statistically significant results were seen for type of KC (BCC vs. cSCC), age at diagnosis and UV-exposed body sites.

Thickness of Actinic Elastosis				TEG		
	Unstandardized Coefficients			Unstandardized Coefficients		
	B	Std. Error	*p*	B	Std. Error	*p*
BCC vs. cSCC	0.128	0.046	0.006	0.446	0.133	<0.001
Age at diagnosis	0.009	0.002	<0.001	0.024	0.005	<0.001
UV-exposed body site	0.266	0.040	<0.001	0.968	0.115	<0.001

## Data Availability

The data presented in this study are available on request from the corresponding author.
